# A comparison of supplemental calcium soap of palm fatty acids versus tallow in a corn-based finishing diet for feedlot steers

**DOI:** 10.1186/s40781-015-0053-5

**Published:** 2015-06-15

**Authors:** Crystal M. Warner, Sahng-Wook Hahm, Shawn L. Archibeque, John J. Wagner, Terry E. Engle, Ivette N. Roman-Muniz, Dale Woerner, Mark Sponsler, Hyungchul Han

**Affiliations:** Department of Animal Sciences, Colorado State University, 80523 Fort Collins, CO USA; Colorado Corn Growers Association, 80631 Greeley, CO USA

**Keywords:** Beef, Fatty acids, Rumen bypass fat

## Abstract

Rumen bypass fat is commonly added to increase energy intake in dairy cattle. The objective of this study is to examine the addition of rumen bypass fat during finishing period on performance and carcass characteristics in grain fed steers. This study was conducted as a completely randomized block design with 126 cross-bred steer calves (initial BW 471.5 ± 7.5 kg) randomly assigned to pens with 9 steers/pen (n = 7 pens/treatment). Each pen was randomly assigned to one of two treatment groups; rumen bypass fat treatment (CCS, calcium soap of palm fatty acids) and control diet (CT, tallow). The diets were formulated to be isonitrogenous and isocaloric. Animals were fed twice daily at 110 % of the previous daily *ad libitum* intake. Blood from each sample was taken from the jugular vein. Muscle and adipose samples were collected from the *longissimus dorsi* regions. Feedlot performance and carcass characteristics were assessed. To examine adipogenic gene expression, quantitative real-time PCR was completed. Steers fed the CT had a greater level of performance for most of the parameters measured. The CT group had greater DMI (*P* < 0.05) and tended to have greater ADG (*P* < 0.10). Marbling score (*P* < 0.05) and quality grade (*P* < 0.05) were greater for steers fed the CT diet than those fed CCS. The longissimus muscle area tended to be greater (*P* < 0.10) in steers fed CT (87.60 cm^2^) than those fed CCS (84.88 cm^2^). The leptin mRNA expression was down-regulated (*P* < 0.05) in adipose tissue of steers fed a CCS when compared to those fed CT. These data suggest that calcium soap of palm fatty acids can be added to finishing diets without significant reduction in final body weight, although there may be modest reductions in marbling and quality scores.

## Background

Currently in the beef industry, the majority are fed high concentrate diets, which is ideal for rapid intramuscular adipose deposition, a characteristic desired for better meat quality [1]. Ruminant fat is an important energy source consisting of predominantly saturated fatty acids (SFA), palmitic acid (C16:0) and stearic acid (C18:0), and polyunsaturated fatty acid (PUFA) [2]. Naturally occurring *trans* fatty acids found in animal products are conjugated linoleic acids (CLA) and vaccenic acid (18:1, *trans*-11) [3]. They are produced from PUFA via ruminal biohydrogenation and are known to be beneficial to human health [4]. Ruminant fat has a lower PUFA: SFA ratio than non-ruminant fat [5]. Saturated fatty acids may have negative health implications, such as increased serum low-density-lipoprotein (LDL) cholesterol concentration and increase risk of coronary heart disease in human [6]. Thus, nutritional strategies that increase of PUFA levels in beef cattle are required to meet the demands of beef consumer. There has been interest over the past years to increase unsaturated fatty acids (UFA) levels in beef. With aims to decrease the SFA concentration of beef intramuscular adipose tissues, research is being conducted in order to minimize the SFA concentration and increase the UFA concentration in ruminant products while maintaining typical, cost efficient, grain-based feedlot finishing diets. One experimental method includes the supplementation of rumen-protected fats. They are insoluble lipids that bypass the rumen and are utilized as an energy source upon absorption in the small intestine. Fat supplementation was originally used to meet the high energy requirements of high producing dairy cattle [7]. Unsaturated fatty acids are also more readily digestible than SFA; therefore increased UFA absorption may improve production efficiency. An increase in milk yield has also been reported by previous study in cows supplemented with 4 % bypass fat [8]. The increased milk yield with supplemental bypass fat may be ascribed to an enhancement in energy balance. Corn based diet with protected fat also showed higher digestibility of ether extracts than corn based diet in beef cattle [9]. Moreover, long-chain omega-3 fatty acids have been correlated to improved reproductive efficiency and carcass characteristics of ruminant animals [10, 11]. According to previous studies, UFA has been found in higher concentration in intramuscular fat of grass-fed beef cattle than in grain-fed [5, 12]. Alternatively, research indicates that grain-fed beef has a greater SFA concentration [13] and a lesser PUFA: SFA ratio than grass-fed beef [14]. Therefore, calcium soap of palm fatty acids supplementation may be an alternative feeding strategy when fresh pasture is not available.

By the inclusion of calcium soap of palm oil fatty acids, UFA are protected from the harsh environment of the rumen allowing them to escape microbial fermentation and biohydrogenation, therefore entering the small intestine as unsaturated and being more efficiently utilized by the ruminant [8]. Through extrapolation of bypass fats efficiency in dairy cattle, it is hypothesized that calcium soap of palm fatty acids supplementation high in UFA during finishing period will increase the circulating UFA in blood and consequently deposition into intramuscular meat in steers fed high corn-based finishing rations. The objective of this study was to examine the influence of feeding calcium soap of palm fatty acids on meat fatty acid composition in grain fed beef steers during finishing period.

## Methods

### Animal care and procedure

A total of 126 cross-bred steer calves (initial BW 471.5 ± 7.5 kg) were housed at Southeastern Colorado Research Center (SECRC, Lamar, CO, USA). The Institutional Animal Care and Use Committee (IACUC) approved the care and use of animals for Colorado State University for this trial. Steers arrived at SECRC 60 days prior to trial commencement and offered *ad libitum* access to alfalfa hay for back grounding. Steers were processed for ear tags, initial body weight and rectal temperatures were recorded. Steers were ranked by body weight and housed 9 steers/pen (n = 7 pens/treatment). Following the 60 day adaptation period, each pen was randomly assigned to one of two treatment groups; rumen bypass fat (CCS, corn based finishing ration with Megalac R) or control diet (CT, corn based finishing ration with tallow) (Table 1). The CCS diet was formulated to be isocaloric and isonitrogenous. The calcium soap of palm fatty acids (Megalac-R, Arm & Hammer Animal Nutrition, NJ, USA) was included in the CCS diet at the expense of primarily tallow. Rumen bypass fat supplement was mixed with total mixed ration (TMR) at 4.305 % DM. Prior to feeding, Rumen bypass fat was added to the feed processor. Treatments were randomly assigned to pens such that 63 animals (7 pens with 9 head/pen) received same diet. Treatment lasted a total of 60 days. Animals were fed twice daily (approximately 7 am and 5 pm) at an estimated 110 % of the previous daily *ad libitum* intake. Feed bunks were cleaned and orts collected on a weekly basis before morning feeding. Dry matter content was analyzed. Diet sample was collected weekly following morning feeding and analyzed for proximate analysis. Body weight of steers was recorded on d 0, 30 and 60 (d 0 is dietary treatment commencement).

**Table 1:** Experimental diet on DM basis

	Treatment	
Ingredient Basis: % of DM	CT	CCS^1^
Corn Silage 50 % Gr	9.78	9.78
Corn Grain Flaked	75.56	76.62
Distillers Gr. + Soluble	4.56	4.23
Corn Steep	3.00	3.00
Urea	1.22	1.22
Tallow	3.79	-
Limestone	1.47	0.23
Potassium Chloride	0.19	0.21
Salt	0.25	0.25
Trace mineral	0.11	0.16
Megalac-R		4.30
Analyzed Nutrients		
Dry matter, % of as-fed	71.83	71.83
Crude protein	13.40	13.40
Non-protein nitrogen	3.41	3.41
Neutral detergent fiber	12.23	12.23
Forage NDF^2,3^	4.00	4.00
NEm^3^, Mcal/cwt DM	98.07	98.07
NEg^3^, Mcal/cwt DM	66.87	66.87
Ether extract	7.03	7.03
Calcium	0.74	0.74
Phosphorus	0.35	0.35
Potassium	0.70	0.70
Magnesium	0.20	0.20
Sulfur	0.20	0.20
Vitamin A^3^, IU/cwt DM	1000	1000
Vitamin E^3^, IU/cwt DM	15	15
Monensin^3^, g/ton DM	30	30
Tylosin^3^, g/ton DM	10	10

^1^ Diets were formulated to be isocaloric and isonitrogenous

^2^Neutral detergent fiber from the forage components of the diet

^3^Theoretical values

### Blood collection

Blood samples were collected on d 0, 30 and 60. Approximately 12 mL of blood was taken from the jugular vein using 20 gauge, 1½ inch vacutainer needles at each collection for fatty acid analysis. Serum from each sample was collected and stored in −80 °C for fat determination. Steers were transported and harvested at commercial slaughterhouse (JBS Swift Slaughterhouse, Greeley, CO, USA) on d 62 and were slaughtered according to industry-accepted procedures.

### Animal harvest and carcass evaluation

Upon slaughter, carcasses were weighed to obtain hot carcass weight (HCW). Muscle and adipose tissue samples were dissected from the *longissimus dorsi* region, flash frozen and stored at −80 °C for subsequent pulverization and fatty acid determination. Following the carcass-chilling period, carcass quality characteristics were assessed according to USDA standard techniques by trained Colorado State University personnel; marbling, quality grade, fat thickness, longissimus muscle (LM) area, KPH (kidney, pelvic, and heart fat), preliminary yield grade, final yield grade, and dressing percent.

### Fatty acid composition analysis

Three steers were randomly selected from each experimental unit (pen). The *longissimus dorsi* region tissue were pulverized using a Waring blender. Blood serum samples were pooled for each experimental unit. Total lipid from blood serum and intramuscular adipose was extracted using the procedure outlined by Folch et al. [15] followed by methylation as described by Morrison et al. [16]. Fatty acid methyl esters (FAME) were determined by gas chromatography (Hewlett-Packard, 6890 series II, CA, USA) equipped with a SP-2560 fused silica capillary column (100 m × 0.25 mm i.d.; Supelco Inc., PA, USA), with a series 7683 injector and flame ionization detector. Helium was used as the carrier gas with a flow rate of 2.1 mL/min. Fatty acids were identified by comparing the retention times displayed on the chromatographs to that of reference standards.

### RNA isolation and quantitative real-time PCR (qRT-PCR)

Total RNA from frozen adipose tissues was isolated with TRIzol reagent (Invitrogen, NY, USA). Isolated RNA was further purified by using RNeasy Mini Kit (Quiagen, CA, USA) with RNase-free DNase (Quiagen, CA, USA) and quantified by use of a Nanodrop spectrophotometer (Thermo Fisher Scientific, DE, USA). Synthesis of cDNA was performed using iScript cDNA synthesis kit (Bio-Rad, CA, USA). Real-time PCR was performed for the targets leptin, lipoprotein lipase (LPL), fatty acid synthase (FASN) and stearoyl-CoA desaturase (delta-9-desaturase, SCD) mRNA and housekeeping glyceraldehydes-3-phosphate dehydrogenase (GAPDH) mRNA (Table 2). Real-time PCR was conducted using the LightCycler480 PCR system (Roche Applied Sciences, IN, USA) with 384-well plates. The PCR cycle conditions were as follows: 95 °C for 3 min, 40 cycles of 95 °C for 30 sec, 61 °C for 30 sec, and 72 °C 15 sec followed by a melt curve analysis to confirm amplification of single cDNA products. The Ct values were obtained from the Lightcycler software (Roche Applied Sciences, IN, USA) and adjusted for GAPDH Ct values for each sample to determine △Ct and relative expression of the target mRNA. Data were presented as 2^-△△Ct^ [17].

**Table 2:** Real-time PCR primer sequences

Target gene	Primer sequence	Accession #
GAPDH	F: GATTGTCAGCAATGCCTCCT	NM_001034034.2
	R: GGTCATAAGTCCCTCCACGA	
Leptin	F: TGTGGCTTTGGCCCTATCTG	NM_173928.2
	R: CGGACTGCGTGTGTGAGATG	
Lipoprotein lipase	F: ATACACCAACCAGGCCTTCG	NM_001075120.1
	R: GCTTTGCCAAGTTTCAGCCA	
Fatty acid synthase	F: CTGCCGAAGACAGGGATTGT	NM_001012669.1
	R: TGTACAGCTTCTGCTGGTGG	
Stearoyl-CoA desaturase	F: CCTGTGGAGTCACCGAACC	NM_173959.4
	R: CAAAAACGTCATTCTGGAACGC	

### Statistical analysis

This study was conducted as a completely randomized block design with repeated measures, consisting of two treatment groups with 63 animals per treatment, divided into 7 pens. In order to minimize the effect of initial body weight, steers were assigned into 1–7 weight blocks based off of body weight at d 0. Rumen bypass fat treatment was randomly assigned into 7 pens, while the other 7 were fed the CT diet. Analysis of Variance (ANOVA) was performed on feedlot performance, fatty acid composition and carcass data were analyzed on a pen mean basis using PROC GLM of Statistical Analysis Software (SAS Institute, Inc., NC, USA). The model includes treatment as fixed effect and pen as random effect for USDA quality and yield grades, incidence of morbidity and mortality and fatty acid profile analysis. The treatment difference was detected using a modified students test. Responses are reported using LS means ± standard error. Significance was determined at *P* < 0.05 and tendency at *P* < 0.10.

## Results and discussion

Feedlot performance and carcass characteristics are presented in Table 3. Steers fed the CT diet had greater DMI (10.14 kg vs. 8.77 kg; *P* < 0.02) than those fed CCS diet. Average daily gain tended to be greater in CT steers when compared to CCS fed steers (1.64 kg vs. 1.25 kg; *P* < 0.07). The smell and taste of calcium soap of palm fatty acids may influenced the palatability of the whole diet with decreased total DMI. The ADG was not increased because of depressed DMI. These results are in agreement with previous research [9] in which decreases in ADG was observed in cattle fed corn based diet with rumen protected fat. It has been also reported that steers fed rumen-bypass PUFA source (Megalac R) had decreased mean DMI and tended to have reduced ADG [18]. Although DMI decreased in CCS steers, the final body weight and gain:feed ratio were not significantly different between CT and CCS steers.

**Table 3:** Feedlot performance and carcass characteristics of steers fed CT (tallow) or CCS (calcium soap of palm fatty acids) diet

	Treatment			
Parameter	CT	CCS^1^	SE	*P* <
Initial BW, kg	471.8	471.4	7.5	0.29
Final BW, kg	570.1	546.6	8.1	0.15
ADG^2^, kg	1.640	1.253	0.07	0.07
DMI^3^, kg	10.140	8.772	0.36	0.02
Gain: Feed	0.162	0.143	0.01	0.14
HCW^4^, kg	360.47	345.8	7.44	0.19
Marbling score^5^	402.9	374.1	8.89	0.04
Quality grade^6^	384.2	362.7	5.50	0.02
Dressing, %	60.67	60.63	0.24	0.90
Adjusted fat thickness, cm	1.035	0.997	0.08	0.73
LM area, cm^5^	87.60	84.88	1.10	0.10
Yield grade	3.019	2.982	0.08	0.74

^1^Diets were formulated to be isocaloric and isonitrogenous

^2^ADG, average daily gain

^3^DMI, dry matter intake

^4^HCW, hot carcass weight

^5^Practically Devoid = 100; Traces = 200; Slight = 300; Small = 400; Modest = 500; Moderate = 600; Slightly Abundant = 700; Moderately Abundant = 800; Abundant = 900

^6^Standard = 200; Select = 300; Choice = 400; Prime = 500

Hot carcass weight was not significantly different between treatments (*P* < 0.19). Marbling score (*P* < 0.05) and quality grade (*P* < 0.05) were greater for steers fed the CT diet than those fed CCS. The *longissimus dorsi* area tended to be greater (*P* < 0.10) in steers fed CT (87.60 cm^2^) than those fed CCS (84.88 cm^2^). The effects of rumen bypass fat on feedlot performance and carcass characteristics were in favor of the CT diet group. Decreased feed intake and BW gain coincides with the tendency (*P* = 0.10) for a lesser *longissimus dorsi* area in the CCS group compared to CT group. Marbling score and quality grade were greater for the CT than CCS. This may be attributed to increased intramuscular fatty acid deposition in steers fed the CT diet as a result of greater feed intake and subsequently greater weight gain. Carcass characteristics were statistically analyzed as categorical data assuming binomial distribution; quality grade for all steer were either select or choice and either had slight or small marbling scores. However, no treatment effects were detected for carcass evaluation.

Numerically, there was an increase in UFA in CCS group in blood and muscle, although it is not statistically significant (Tables 4 and 5). No significant effects of CCS group on fatty acids composition in *longissimus dorsi* muscle tissue were found (Table 4). The linoleic acid (C18:2 t) in blood was tended to be greater in CCS group when compared to CT group (Table 5). Day intervals in Table 5 were not expressed for fatty acids where there was no treatment by time interactions. However, significant treatment by time interactions were observed between treatment groups for palmitoleic acid (C16:1) and oleic acid (C18:1 c9) in fatty acid composition of serum. At day 60, the CCS diet had a significant decrease in palmitoleic acid. Alternatively, at day 60, the CCS diet was significantly increased in oleic acid than was the CT diet. This tradeoff may be explained by the high C18:1 concentration in the CCS treatment diet. Due to the treatment by time interaction seen in the blood serum at day 60, a longer feeding period may be necessary for increased effects of rumen protected fat on the fatty acid composition of blood serum, which may result in alteration of muscle fatty acid composition. As fatty acids enter the circulatory systems, free fatty acids are transported in the blood bound to albumin while other lipids are transported packaged as lipoproteins and delivered to tissues [19]. Continued increase in circulating fatty acids and thus increased delivery to tissues, will potentially produce a greater content of these fatty acids in the muscle tissue. Moreover, higher proportion of CLA was observed in CCS groups. CLA are isomers of linoleic acid (18:2) with conjugated double bonds, most notably *cis*-9, *trans*-11-octadecadienoic acid (*c*9, *t*11-18:2) [20]. Research has indicated that CLA have anticarcinogenic effects [21, 22], enhance immune response [23], reduce LDL plasma cholesterol levels, and suppress cholesterol-induced atherosclerosis [24, 25]. Although the results of fatty acids composition from the present study do not reflect the effects on human health, we could expect that the quality of fatty acids composition in adipose tissues of beef cattle would be improved by the manipulation of UFA composition through the supplementation of calcium soap of palm fatty acids.

**Table 4:** Effects of CT (tallow) or CCS (calcium soap of palm fatty acids) diet on fatty acid composition of beef longissimus dorsi muscle (% of total fatty acids reported)

	Treatment			
Fatty acid	CT	CCS^1^	SE	*P* <
C14:0	3.67	3.44	0.15	0.29
C14:1	0.27	0.26	0.03	0.82
C15:0	0.64	0.59	0.05	0.74
C16:0	25.30	25.45	0.53	0.42
C16:1 c9	1.11	0.85	0.36	0.41
C17:0	1.60	1.46	0.17	0.67
C18:0	20.07	19.49	1.02	0.45
C18:1 c11-15	6.37	6.06	0.42	0.65
C18:1 c9	34.68	36.14	0.65	0.76
C18:1 t (total)	3.71	3.53	0.21	0.84
C18:2 Total	1.77	1.76	0.10	0.44
C18:2 t^2^	0.59	0.68	0.07	0.29
C18:3 n-3	0.22	0.24	0.01	0.74
C20:1 c11	0.18	0.14	0.02	0.38
C22:5 n-3	0.02	0.02	0.01	0.87
Saturated	51.09	50.30	0.18	0.86
Unsaturated	48.91	49.70	0.19	0.84
MUFA	46.31	47.00	0.12	0.68
PUFA	2.60	2.70	0.08	0.21
Sat.: Unsat.^3^	1.04	1.01	0.02	0.41

^1^Diets were formulated to be isocaloric and isonitrogenous

^2^Includes C18:2 c9 t11, C18:2 t10 c12, C18:2 c11 t13, and C18:2 tt

^3^Total saturated fatty acid to total unsaturated fatty acid ratio

**Table 5:** Effects of CT (tallow) or CCS (calcium soap of palm fatty acids) diet on fatty acid composition of serum (% of total fatty acids reported)

	Treatment			*P* <		
Fatty acid	CT	CCS^1^	SE	Trt	Time	Trt x Time
C14:0	0.92	0.91	0.09	0.62	0.29	0.76
C14:1	0.63	0.65	0.08	0.95	0.18	0.83
C15:0	1.18	1.20	0.05	0.58	0.14	0.93
C16:0	21.70	20.30	0.82	0.24	0.27	0.85
C16:1^2^	2.58	2.35	0.12	0.21	0.21	0.02
0d	2.39	3.06	0.20	0.18	-	-
30d	2.68	2.02	0.13	0.07	-	-
60d	2.65	1.94	0.07	0.03	-	-
C17:0	2.48	2.37	0.13	0.57	0.00	0.68
C17:1	0.86	0.72	0.06	0.09	0.04	0.87
C18:0	14.19	14.12	0.73	0.96	0.84	0.87
C18:1 c9^2^	5.92	6.77	0.65	0.37	0.00	0.04
0d	4.50	4.08	0.72	0.20	-	-
30d	5.62	7.61	0.61	0.05	-	-
60d	4.70	8.02	0.44	0.01	-	-
C18:1 t (total)^3^	2.48	2.54	0.15	0.87	0.08	0.89
C18:2 t (total)^4^	3.37	3.39	0.04	0.06	0.88	0.71
C18:2	38.67	39.39	1.37	0.32	0.06	0.94
C18:3 n-3	1.17	1.09	0.13	0.78	0.01	0.95
C20:1 c11	2.98	2.72	0.48	0.60	0.18	0.68
C22:5 n-3	1.85	1.69	0.32	0.61	0.01	0.79
Saturated	40.47	38.90	1.09	0.29	0.25	0.76
Unsaturated	59.52	61.10	1.21	0.21	0.12	0.68
MUFA	14.46	15.54	0.75	0.68	0.02	0.29
PUFA	45.06	45.56	1.41	0.58	0.14	0.96
Sat.: Unsat.^5^	0.68	0.62	0.021	0.23	0.03	0.54

^1^Diets were formulated to be isocaloric and isonitrogenous

^2^Trt × time interaction (*P* < 0.05). Fatty acids were indicated by each day of blood samples collection

^3^Includes C18:1 t-10 and t-11

^4^Includes C18:2 c9,t-11; t-10, c-12; c-9, c-11; t-9, t-11

^5^Total saturated fatty acid to total unsaturated fatty acid ratio

The low rumen pH caused by high concentrate diets typically fed to feedlot cattle may contribute to minimal changes of fatty acid profile. Due to the differences in feed formulation of dairy versus feedlot cattle, bypass fat function differs at the opposing rumen pH. High forage concentrations of dairy cattle yield an increased pH, while the higher concentrate diets of feedlot cattle decreases rumen pH. Rumen bypass fats are more stable and work optimally at a normal rumen pH of 5.5–6.5 [26]. It has been reported that ruminal fluid pH was 5.8 in cattle fed corn based diet with protected fat for 41 days [9]. As pH decreases in the rumen, calcium soaps more readily dissociate leaving the free fatty acids susceptible to biohydrogenation into their saturated form for subsequent absorption. Therefore by increasing rumen pH more fatty acids may stay intact with the calcium ion, escaping biohydrogenation and dissociate in the abomasum and duodenum for subsequent digestion. Future studies under optimized ruminal pH conditions are needed to better understand the rumen bypass fat and UFA production mechanism in grain finishing beef cattle.

To obtain a clear understanding for the carcass characteristics results, the mRNA expression of genes involved in lipogenesis was examined in adipose tissue. The down-regulation of leptin mRNA was observed in steers fed a CCS diet when compared to those fed CT diet (*P* < 0.05) (Table 6). The SCD mRNA expression tended (*P* = 0.07) to be less in steers fed a CCS diet when compared to those fed CT diet. There were no significant differences between the treatments in LPL (*P* = 0.92) and FASN (*P* = 0.32) mRNA expression. Leptin, a protein hormones synthesized and secreted by adipocytes in white adipose tissue, may be an indicator of marbling, back fat depth, and quality grade of feedlot cattle [27]. Moreover, bovine leptin gene is related to marbling [28]. In this study, mRNA expression of leptin in adipose tissue was reduced by calcium soap of palm fatty acids supplementation. This is reasonable in that steers fed the CT diet had increased carcass weight and marbling scores. Greater marbling score may induced greater leptin production in steers fed a CT diet. This supposition is reinforced by the positive correlations existing between marbling score and serum leptin in cattle as reported by Geary et al. [29]. Reduced leptin expression in adipose tissue would decrease circulating concentration of leptin, therefore, it seems reasonable that marbling score could also decrease.

**Table 6:** Altered mRNA expression of genes involved in lipid metabolism of steers adipose tissue

Gene	CT^1^	CCS^2^	*P* <
Leptin	1.17 ± 0.08	0.82 ± 0.13	0.04
LPL	1.75 ± 0.24	1.69 ± 0.55	0.92
FASN	2.12 ± 0.75	1.19 ± 0.49	0.32
SCD	1.17 ± 0.12	0.68 ± 0.21	0.07

The data are expressed as the means ± S.E

Lipoprotein lipase, LPL; Fatty acid synthase, FASN; Stearoyl-CoA desaturase, SCD

^1^CT, corn based diet-tallow

^2^CCS, corn based diet-calcium soap of palm oil fatty acids

Other genes related to lipogenesis, including LPL, FASN and SCD were observed. LPL hydrolyzes triglyceride-rich lipoproteins to make NEFA for uptake in peripheral tissues [30]. FASN regulates lipid biosynthesis from NADPH and acetyl-CoA in adipose tissue [31]. LPL and FASN are important enzymes in adipocyte lipogenesis and have been found to have greater mRNA expression in the subcutaneous fat than in the intramuscular fat tissue [32]. This may explain the similar levels of LPL and FASN expression observed from the tissue between treatments groups. While marbling score of carcass did statistically differ between groups, similar fat thickness was observed in the present study. Stearoyl-CoA desaturase 1 is an enzyme involved in the catalyzing the desaturation of saturated fatty acids to monounsaturated fatty acids (MUFA) [33]. It has previously been reported that increasing n-3 PUFA composition in the diet caused a significant reduction in SCD expression in subcutaneous adipose tissue of Holstein bulls [34]. It may be explained by the correlation with reduced gene expression levels of lipogenic transcription factor (sterol regulatory element binding protein 1c, SREBP1c) in adipose tissue. These results are in agreement with the findings of the current study as CCS supplementation showed decrease in SCD mRNA expression as compared to steers fed CT diet. Moreover, alpha-linolenic acid (C18:3 n-3) inhibited SCD gene expression in the adipocytes of mice [35]. However, MUFA composition of *longissimus dorsi* muscle was not affected by CCS supplementation. In some studies, there was no correlation between Δ9-desaturase index and SCD gene expression in subcutaneous fat [36], and the Δ9-desaturase index does not correctly reflect the enzyme activity in cattle [37]. Similarly, Wynn et al. [38] observed no effect of Megalac supplementation on SCD mRNA expression level in sheep. Further study is needed to clarify whether there is difference in SCD expression depends on supplementation methods and periods.

## Conclusions

In conclusion, these data suggest that calcium soap of palm fatty acids can be added to finishing diets without significant reduction in final body weight, although there may be modest reductions in marbling and quality scores. Few significant differences were seen in the muscle and serum fatty acid composition. Adjustment of finishing diet formulation in favor of a more basic pH may increase PUFA in intramuscular adipose, although this may result in decreased ADG and therefore a longer time requirement to reach slaughter weight. Potential future research to consider for a significantly increase UFA content include feeding rumen bypass fat for an extended time period as opposed to the 60 days fed in this trial.
